# Medical Topography of La Plata

**Published:** 1843-04

**Authors:** 


*Medical Topography of La Plata.*—We are indebted for the following account to a review in the Gazette des Hôpitaux, of a pamphlet by Adolphus Brunel, surgeon-major to the corvette La Perle. It is entitled 'Topographical, meteorological, and medical observations made in Rio de la Plata, during the blockade of Buenos Ayres.'

The Rio de la Plata is a great mass of water lying between the 34th and 36th degree of south latitude, which after having received the Parana, the Paraguay, and the Rio-Salado, falls into the Atlantic. Two considerable towns, Buenos Ayres and Monte Video, the capitals of the Argentine and the Eastern Republics, are situated, one on its right, and the other on its left bank. Its shores are pleasantly situated, with a temperate climate, and a very fertile soil. The spring begins in September, the summer in December, and the autumn in March. During the author's stay, the greatest cold was 4+0 of Reaumur,=41° of Fahr; and the greatest heat 24° R.=86 F. The most frequent winds are from the N., the N.E., and the S.E.; they blow with violence, often change, and produce sudden and frequent alterations in the temperature and hygrometric state of the atmosphere.

The people who inhabit these countries may be divided into four principal classes.

First, the Creoles, sprung from the conquerors, to whom may be added all the Europeans and North Americans. Secondly, the Negroes imported from the coast of Africa. Thirdly, the half-castes, formed by the intermixture of Europeans, natives, and negroes. Fourthly, the natives, who may be divided into two classes; those, namely, who have become civilized, and those who, resisting every attempt of the Spaniards to subjugate them, have preserved their independence.

The Indians in the savage state inhabit the Pampas, and the deserts of the great Chaco; and they are divided into tribes of eighty or a hundred families, governed by a chief, who is generally elected. Since the importation of cattle, and particularly of horses, into America, those tribes are no longer cannibals. These Indians are below the middle stature; their head is large, their nose short and flat, and their cheekbones prominent. The projection of the chin is wide, and the chin itself is the only part of the face that has hair; their lips are thin, and their mouth furnished with splendid teeth, which are admirably regular, and remain uninjured to an advanced age. Their chest is broad and prominent, and their limbs well turned; their hands and feet small; their complexion of a pale copper color; their long black hair meets at the top of the head, and they sometimes let it hang down; their hearing is very fine, and their sight piercing; their constitution is robust, they are bold riders, and never go but on horseback. They are distrustful, thievish, greedy, and cruel; drunkenness is their ruling passion; they are naturally lazy, and oppress their women with work. Their chief nourishment consists of horse-flesh, which they eat almost raw, and when it has reached a certain degree of putrefaction; at other times they dry the meat, and reduce it to a powder, mix it with plenty of salt, and make it into a paste. They use maize as food, and also make a fermented drink from it. The coagulated blood of a colt, kneaded with maize flour and salt, is one of their tit-bits. At present, the incursions of these savages into the different provinces of La Plata are less frequent than formerly; yet from time to time they plunder the farms and carry off the cattle. They make war with ferocity, massacreing the men, and sparing only the women, and sometimes the children.

This part of America is exempt from the morbid scourges which devastate other countries. The plague of the East, the yellow fever of the Antilles, the cholera, typhus, and intermittent fevers, are not seen there. Among common diseases are catarrhs, sore throats, croup, hooping-cough, pleurisy, and pneumonia, all connected with the rapid variations of temperature. Phthisis is also very destructive there. Diseases of the skin are very frequent; small-pox, measles, and scarlatina, are dominant epidemics. Caries of the teeth is endemic, and appears very early; girls, in particular, of sixteen or seventeen, have none but decayed teeth. The ravages of small-pox are frightful, especially among the native savages, whole tribes of whom it has often destroyed. The slightest wound, and frequently the mere transition from heat to cold, is sufficient to produce immediate tetanus. The negroes and Europeans are more usually attacked by it than natives. The *gauchos*, or half-caste shepherds, often succeed in treating this terrific disease by wrapping the patient in a sheep-skin recently flayed. Hepatitis and dysentery are common in the towns, and syphilitic diseases in the country. Goitre is endemic; and the negroes are subject to tubercular lepra.—*New York Lancet.*

